# Observation of intermolecular Coulombic decay and shake-up satellites in liquid ammonia

**DOI:** 10.1063/4.0000151

**Published:** 2022-07-28

**Authors:** Hanns Christian Schewe, Eva Muchová, Michal Belina, Tillmann Buttersack, Dominik Stemer, Robert Seidel, Stephan Thürmer, Petr Slavíček, Bernd Winter

**Affiliations:** 1 Institute of Organic Chemistry and Biochemistry of the Czech Academy of Sciences, Flemingovo nam.2, 16610 Prague 6, Czech Republic; 2 Department of Physical Chemistry, University of Chemistry and Technology, Technická 5, Prague 6, 16628, Czech Republic; 3 Fritz-Haber-Institut der Max-Planck-Gesellschaft, Faradayweg 4-6, 14195 Berlin, Germany; 4 Operando Interfacial Photochemistry, Helmholtz-Zentrum Berlin für Materialien und Energie, Hahn-Meitner-Platz 1, 14109 Berlin, Germany; 5 Humboldt-Universität zu Berlin, Institut für Chemie, Brook-Taylor-Str. 2, 12489 Berlin, Germany; 6 Department of Chemistry, Graduate School of Science, Kyoto University, Kitashirakawa-Oiwakecho, Sakyo-Ku, Kyoto 606-8502, Japan

## Abstract

We report the first nitrogen 1s Auger–Meitner electron spectrum from a liquid ammonia microjet at a temperature of ∼223 K (–50 °C) and compare it with the simultaneously measured spectrum for gas-phase ammonia. The spectra from both phases are interpreted with the assistance of high-level electronic structure and *ab initio* molecular dynamics calculations. In addition to the regular Auger–Meitner-electron features, we observe electron emission at kinetic energies of 374–388 eV, above the leading Auger–Meitner peak (3a_1_^2^). Based on the electronic structure calculations, we assign this peak to a shake-up satellite in the gas phase, i.e., Auger–Meitner emission from an intermediate state with additional valence excitation present. The high-energy contribution is significantly enhanced in the liquid phase. We consider various mechanisms contributing to this feature. First, in analogy with other hydrogen-bonded liquids (noticeably water), the high-energy signal may be a signature for an ultrafast proton transfer taking place before the electronic decay (proton transfer mediated charge separation). The *ab initio* dynamical calculations show, however, that such a process is much slower than electronic decay and is, thus, very unlikely. Next, we consider a non-local version of the Auger–Meitner decay, the Intermolecular Coulombic Decay. The electronic structure calculations support an important contribution of this purely electronic mechanism. Finally, we discuss a non-local enhancement of the shake-up processes.

## I. INTRODUCTION

The introduction of liquid-phase photoemission (PE) spectroscopy based on liquid microjet (LJ) technology makes it possible to investigate electronic decay processes in volatile liquids.^1,2^ Considerable focus has recently been given to the phenomena emerging in the condensed phase, such as the intermolecular decay processes and ultrafast reactions between the highly excited or ionized molecules. The majority of previous studies involve liquid water and aqueous solutions, and water can, thus, serve as a reference system for other liquids. In the present study, we present the very first Auger–Meitner^3–5^,^80^ electron spectrum of liquid ammonia, providing the first account on the electronic relaxation processes following the core-level ionization.

We begin by discussing purely electronic processes. In the liquid phase, the dominant electronic decay process is still the Auger–Meitner process in which an electron from a valence state fills the core-level vacancy, and the energy released is used to eject another valence electron from the same molecule. The liquid environment acts as a polarizable continuum and, thus, shifts the energy of this process by several electron volts as compared to the gas-phase. The more dramatic effect of the condensed phase is represented by the so-called interatomic or intermolecular Coulombic decay (ICD). It is a non-local electronic relaxation process taking place in weakly bound systems such as rare-gas complexes or hydrogen-bonded aqueous-phase systems.^6–8^ Upon ICD, an initially core or inner-valence ionized atom or molecule electronically decays, and the released energy ionizes a *neighboring* entity via Coulombic electron interactions. The emitted (autoionized) electron is referred to as an ICD electron. Here, we specifically explore the possible role of ICD following core-level ionization in a different solvent than water and expand on previous works on ammonia in the aqueous phase.^9^

The ICD phenomenon was first predicted for hydrogen-bonded clusters, including water clusters, upon inner-valence ionization.^10^ However, the first systematic studies of ICD following inner-valence ionization were done for prototypical rare-gas aggregates and clusters. In those experiments, the interpretation of the spectra is not complicated by the nuclear dynamics of the constituent atoms, and furthermore, valence ionization does not lead to (local) Auger–Meitner processes, which would compete with the non-local processes.^7^ Yet, subsequent studies have demonstrated the occurrence of ICD upon core-level ionization,^8^ with a relatively high probability (about 1%), as documented, e.g., for argon clusters.^11^

**FIG. 1.**
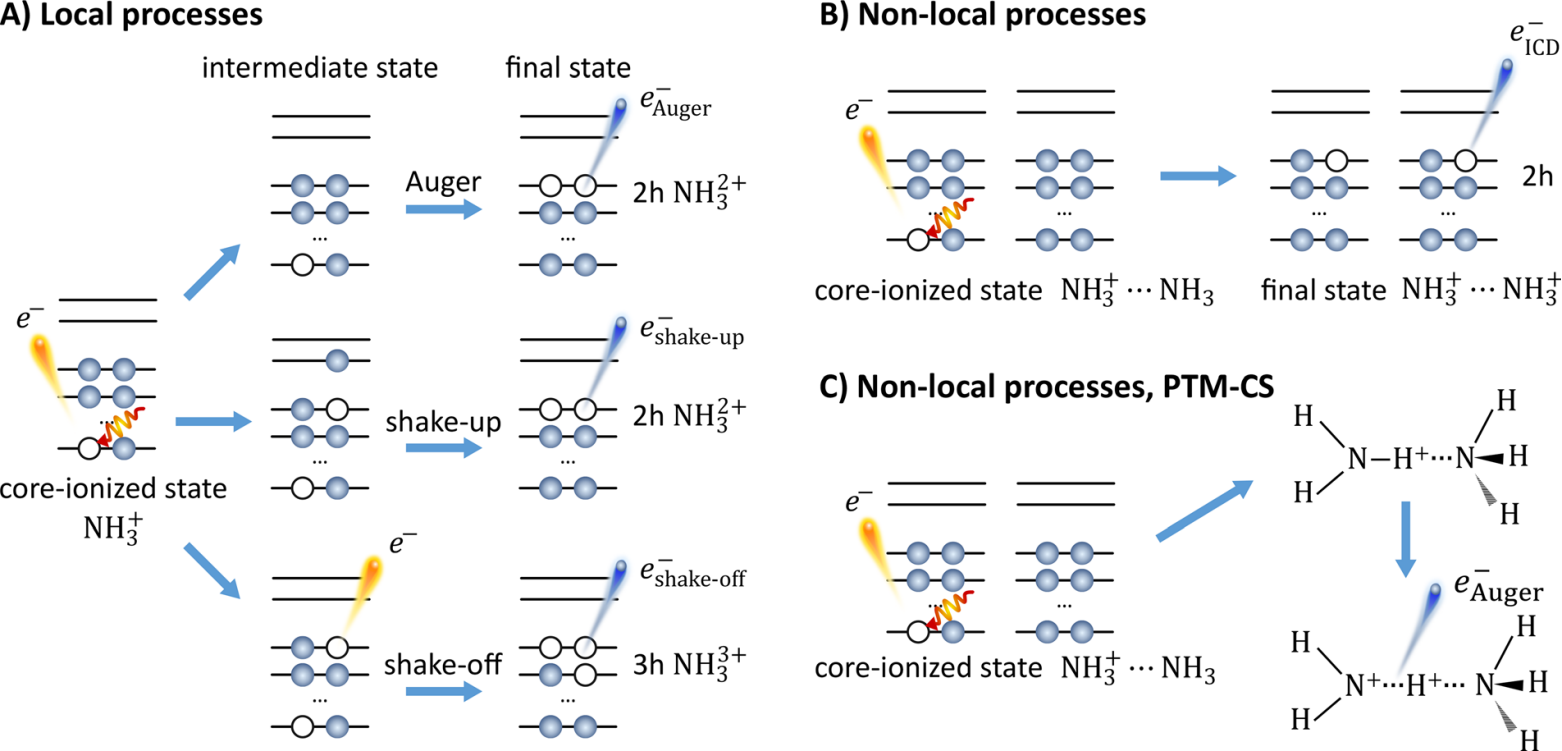
A sketch of ultrafast decay channels following N 1s core ionization in ammonia. (a) (Local) processes considered for the monomer: Normal autoionization (top), shake-up autoionization (center) after additional valence excitation, and shake-off autoionization (bottom) after additional valence ionization. (b) (Non-local) ICD process in ammonia dimer. The energy released from the decay couples to a neighboring molecule which is ionized, thus distributing the charge over two entities. Here, NH_3_^+^ ··· NH_3_^+^ refers to the ground-state structure implying pure ICD. (c) Sketch of a (non-local) PTM-CS process in the ammonia dimer. After core-level ionization of the proton donor, the proton-transfer reaction takes place. The Auger–Meitner electron is emitted from the Zundel-analogue structure. Note that such a process is unlikely to occur in liquid ammonia and is just added for illustration purposes.

The electronic relaxation processes in molecular liquids are complicated by the fast nuclear dynamics, noticeably the ultrafast intermolecular reactions. This has been best documented for water. In the case of O 1s core ionization of liquid water, highly electronically excited H_2_O^+*^ (with excess energy of approximately 540 eV) is formed, and in a subsequent ICD process, a pair of two H_2_O^+^···H_2_O^+^ water cations may form, spatially separated by Coulomb repulsion. The total energy of such a charge-delocalized product is lower than that of H_2_O^2+^ formed in a competing (local) Auger–Meitner process. The local Auger–Meitner and non-local ICD processes can be experimentally disentangled by the appearance of an additional high-kinetic-energy signal contribution from ICD in the O 1s Auger–Meitner electron spectrum of the liquid phase; this signal contribution is absent in the gas-phase spectrum.^1,2^ As mentioned, the relaxation process can be accompanied by nuclear dynamics, in which case, a proton from the initially ionized molecule moves toward a neighboring molecule, as occurring in liquid water.^1^ This process, termed proton-transfer mediated charge separation (PTM-CS),^1,2^ is very fast given the deep core-hole potential and competes with the native (purely electronic) ICD process. There is, thus, a large probability for the electronic decay in a distorted molecular configuration, on the 4-fs timescale of the O 1s core-hole lifetime, and the ‘pure’ ICD process yielding H_2_O^+^···H_2_O^+^ (associated with the water ground-state structure) final product cannot be captured experimentally. Instead, PTM-ICD will lead to [HO···H^+^···OH_2_^+^],^1^ which is experimentally manifested by spectral contributions between the kinetic energy associated with the regular Auger–Meitner decay and the neat ICD channels. Corresponding processes in liquid ammonia are sketched in Fig. 1.

Experimentally, the PTM-CS mechanism is directly revealed by the isotope effect, first demonstrated for liquid water, contrasting light vs heavy water. In this way, the proton-transfer contribution to the non-local decay processes was subsequently demonstrated in various hydrogen aqueous solutions, including hydrogen peroxide, ammonia, and amino acids solvated in water.^1,9,12^ The magnitude of PTM-CS was observed to correlate positively with the hydrogen-bond strength in the liquid state. Arguably, the strongest manifestation of the PTM-CS phenomenon was reported for hydrated NH_4_^+^, where a double-proton transfer was shown to take place.^13^ Transient molecular intermediates, associated with the nuclear dynamics, may also play an important role in radiation chemistry.^14–17^

There is yet another general mechanism to consider, which can alter the energetics of the decay process following core-level ionization. Given sufficient energy input, the initial ionization step can lead to an additional valence excitation (shake-up) or valence ionization (shake-off), from which the subsequent decay (e.g., autoionization process) will proceed under altered conditions (different transient electronic structures);^18^ an illustration of these processes is sketched in Fig. 1(a). Note that the shake-off processes increase the charge state of the species, which considerably reduces the energy of the outgoing electrons via Coulomb attraction. The energetics of the shake-up decay depend on the particular electronic structure, but in general, Auger–Meitner electrons from shake-up states yield higher kinetic energy than the leading Auger–Meitner electron line (highest kinetic energy associated with local decay) due to the screening effect provided by the excited electron in the case of a spectator-type process or due to the lower binding energy of the shake-up electron in the case of a participator-type process. The latter is shown in Fig. 1 and was investigated in our calculations, and spectator-type processes are not.

Water and ammonia are isoelectronic molecules. Liquid water and liquid ammonia both form hydrogen-bonding networks, with rather similar solvation properties, and yet differences in hydrogen-bond strength cause noticeably different dynamics.^9^ Ammonia has a lower propensity for proton transfer even in the ground electronic state, associated with its significantly lower autoionization constant (∼10^−30^ for ammonia vs ∼10^−14^ for water, considering the 1 M standard state). Furthermore, the somewhat weaker, less directional hydrogen bonding in liquid ammonia^13,19^ is expected to slow down the proton transfer in the core-ionized state as well, and thus, the (pure) ICD signal associated with the ground-state geometry is expected to be dominant in liquid ammonia, unlike for water.

The Auger–Meitner spectra of ammonia were previously recorded and interpreted from the gas phase,^20–22^ from large ammonia clusters^23^ — which can be viewed as a solid amorphous state of matter — as well as from solid state.^24^ Auger–Meitner spectra and non-local effects upon core-level ionization were also calculated for small ammonia clusters.^25^ The main difference observed when going from the gas phase to the solid state is a considerable broadening of the peaks derived from the 3a_1_-type molecular orbitals, which are involved in the hydrogen bonding of the molecular crystals. The solid-state spectrum has been analyzed in terms of seven main peaks, similarly as for the free-molecule spectrum, and in both cases, an additional satellite structure at higher kinetic energy was described. The overall spectral shape measured from ammonia clusters and solid state is rather similar, and relevant differences will be addressed in the Results section.

PE studies with liquid ammonia have not been possible until the very recent demonstration of a low-temperature liquid-ammonia microjet applicable for PE experiments. Ammonia is a gas at ambient conditions and must be liquefied and stabilized at a temperature between its boiling point of 240 K (−33 °C) and its melting point of 195 K (−78 °C) prior to injection into a vacuum. If properly temperature-controlled, this approach enables the characterization of the electronic structure of liquid ammonia, including the formation of solvated di-electrons, the electrolyte-to-metal transition upon dissolution of alkali metal, and the electronic properties of ammonia solutions containing benzene molecules.^26–29^ Here, we report the very first Auger–Meitner electron spectrum, associated non-local relaxation channels as well as shake-up processes measured upon N 1s core-level ionization of liquid ammonia. The measured spectrum is then contrasted with the one from the gas phase as well as with previously reported measurements from ammonia clusters.^23^ Our focus lies on the relative roles that electronic decay (ICD, shake-up, and shake-off processes) and nuclear dynamics play in non-local electronic relaxation and, specifically, the effect of weaker hydrogen bonding (compared to neat liquid water) on potential PTM-CS-like processes in liquid ammonia. We also discuss these processes for ammonia as a solute in the aqueous phase.

## II. METHODS

### A. Experiment

A cylindrical LJ of liquid ammonia was generated in a three-step procedure: (i) Gaseous ammonia was condensed in an evacuated borosilicate glass cylinder submerged in a bath of cooled ethanol at −50 °C. After condensing a sufficient volume of pure ammonia (Westfalen, purity 5.0), NaI salt (Merck, purity 99.8%) was added to form solutions of 50 mM concentration. Analogous to LJ-PE studies from aqueous solution, the addition of salt ensures sufficient conductivity to avoid sample charging.^30^ (ii) After preparation, the ammonia solution was transferred into a stainless-steel reservoir contained within an ethanol-cooled bath. The principles of this cryostat unit have been described in Refs. 26 and 27, and the actual setup used in this study is presented in Ref. 29. (iii) Finally, argon gas was pressed with ∼5 bar into the head space of the sample cylinder, which pushed the ammonia solution through a 6-mm diameter stainless steel tube with a polyethylene filter at its end. The LJ was formed by a quartz capillary (inner diameter of ∼100 *μ*m) glued into the outlet of the polyethylene filter.^26^ Laminar flow was observed for 2–4 mm downstream (depending on the pressure in the head space of the liquid ammonia reservoir) before the jet disintegrated into droplets. The droplets (moving from top to bottom) were frozen out inside the catcher unit underneath the quartz capillary at the far end of the chamber, which was submerged in liquid nitrogen from the outside of the vacuum chamber. A second liquid-nitrogen cooled cold trap was placed inside the main chamber to aid in pumping ammonia vapor.

Unlike in our previous liquid ammonia studies, all components of the cryostat unit were electrically decoupled from the grounded apparatus (including the electron analyzer) by insertion of a ceramic flange.^29,31^ The equilibrated liquid ammonia solution, in direct contact with the (metallic) cryostat unit, can be put on the same potential as the apparatus (common ground). Alternatively, a negative bias voltage *U* can be applied to the liquid jet by connecting a power supply (Delta Elektronika ES 0300) to the cryostat with respect to the grounded apparatus. The effect of *U* is to generate an electric-field gradient between the LJ and the electron analyzer (orifice), which accelerates electrons emitted from the liquid surface and uniformly shifts the Auger–Meitner electron spectrum toward higher kinetic energies.^30,32^ Electrons created in the gas phase surrounding the jet experience less acceleration, with the exact magnitude depending on the point of origin in the electric-field gradient between jet and analyzer orifice where molecules are ionized. This rigid spectral shift effectively separates the pure liquid-phase spectral contributions in kinetic energy from the gas-phase ones, see a recent description in Ref. 30. When applying a large enough negative bias voltage, *U* = –50 V in the present study, the gas-phase signal smears out, and an essentially gas-free spectrum can be obtained. Note that gas-phase molecules very close to the liquid surface will always experience the full bias potential, which can cause a noticeable residual broad signal, particularly at high vapor pressure as in the present study. We will apply both methods, grounded jet (method 1) and biased jet (method 2), with the former requiring subtraction of the gas-phase reference spectrum to obtain a liquid-only spectrum. We specifically employ both methods here to rule out introducing artifacts in the spectrum of liquid ammonia. Briefly, method 1 potentially suffers from over- or underestimating the amount of gas-phase signal in the subtraction process, while method 2 includes a significant additional broad background signal from the smeared-out gas signal in the case of the highly volatile ammonia.

Measurements of Auger–Meitner and associated non-local electron spectra from ammonia liquid microjets, at approximately 80 eV above the nitrogen N 1s edge, were performed at the BESSY II synchrotron radiation facility, Berlin, using the SOL^3^PES setup,^31^ equipped with a near-ambient-pressure capable HiPP-2 hemispherical electron analyzer (HEA, Scienta Omicron). The HEA was operated in sweep mode, at a pass energy of 100 eV and an energy step size of 100 meV. The measurements were conducted using 480 eV photon energy from the U49-2_PGM-1 soft x-ray beamline.^33^ The x- focal spot-size was approximately 40 × 60 *μ*m^2^ (horizontal × vertical). Electrons were detected colinear to the linear polarization vector of the horizontally polarized light. The total experimental energy resolution was ∼280 meV, resulting from combined beamline and electron analyzer resolution, set at ∼120 meV and ∼250 meV, respectively.

### B. Calculations

We modeled two aspects of ultrafast decay processes in ammonia: the energetics and intensities of the non-local autoionization processes and proton dynamics in the core-ionized state. The energetics were investigated for an isolated ammonia molecule or an ammonia dimer in the gas phase, and the liquid phase modeled as a polarizable continuum within the non-equilibrium formulation;^34,35^ the dielectric constant was set to 24, and the optical dielectric constant was set to 1.93^36,37^ to match the experimental conditions. We also extended the model to an ammonia trimer and tetramer. For the former, we used both a random non-cyclic structure taken from the molecular dynamics performed for 51 ammonia molecules (as described in Sec. III) and an optimized cyclic ammonia trimer. For the ammonia tetramer, we took a random structure from molecular dynamics. The ammonia dimer and cyclic trimer were optimized at the Møller–Plesset perturbation theory (MP2) level with the aug-cc-pVTZ basis set. The N 1s ionization energies were evaluated as the energy differences between the ground electronic and core-ionized states described by the maximum overlap method (MOM)^38,39^ at the CCSD(T)(coupled cluster with single and double and perturbative triple excitations) or wB97X/D levels with the cc-pVTZ basis on hydrogen atoms and the cc-pCVTZ basis on nitrogen atoms. The absolute energy positions of the Auger–Meitner peaks (on the kinetic energy scale) were calculated analogously as the energy difference between the core-ionized and the doubly ionized state. The energies of the valence-excitation (shake-up) or valence-ionization (shake-off) states were evaluated with the MOM method at the same level of theory. For modeling the shake-up states, the valence electron was excited into the lowest unoccupied orbital (LUMO). In some cases, the convergence of the MOM wave function was rather poor; therefore, the valence electron was promoted to a higher virtual orbital; the energy was then corrected by an approximate Δ*E*, which corresponds to the energy difference of the respective orbitals (∼2 eV). It is important to note that in these cases, the calculated energies should be viewed only as an estimate.

For modeling of Auger–Meitner decay rates, we employed the Feshbach–Fano approach as implemented in the Q-Chem package (version 5.4).^40–42^ The bound part of the wave function in the initial and final state was described at the EOM-CC (equation of motion coupled cluster theory) level, and the Auger–Meitner electrons were described as plane waves. The initial core-ionized states were described at the EOM-IP-CCSD/cc-pVTZ (equation of motion coupled-cluster ionization potential method) level with core-valence separation (CVS-EOM-IP-CCSD),^43,44^ and the final states were described using a double-ionization-potential variant (EOM-DIP-CCSD/cc-pVTZ level).^45–47^ The partial autoionization widths, i.e., the reciprocal value of the lifetime for a given channel as a measure of the intensity of a given channel in the Auger–Meitner spectrum, were calculated in terms of one- and two-body Dyson functions as described in Refs. 40 and 41. These calculations were performed for the ammonia monomer, dimer, trimer, and tetramer in the gas phase.

The reaction events following the core ionization were first modeled for the ammonia dimer in the gas phase with Born-Oppenheimer molecular dynamics. The initial conditions for the classical equations of motion were obtained using path-integral (PI) molecular dynamics (MD) simulations accelerated with a quantum thermostat based on the generalized Langevin equation (the so-called PI+GLE scheme).^48–50^ The scheme allowed to reduce the number of beads to four without compromising the quality of the evaluated nuclear density covering the nuclear quantum effects. The GLE parameters for the simulations were taken from the GLE4MD webpage using a target temperature *T *=* *235 K and parameters Ns=8 and ℏω/kT=20.^48,49^ The temperature was set slightly above the estimated range of temperatures in the sample to facilitate the sampling. The energies and forces in the ground state were evaluated at the BLYP level with the 6–31++G^**^ basis set, using the D3 empirical dispersion.^51^ The total simulation time was 48 ps with a time step of 20 a.u. (∼0.5 fs), and we selected equidistantly 300 geometries from this trajectory for subsequent simulations in the core-ionized states. The energies and forces of the core-ionized states were evaluated with the MOM method, using the wB97X/D functional and the cc-pCVTZ basis set on nitrogen atoms and the cc-pVTZ basis set on hydrogen atoms. The simulations on the core-ionized potential energy surfaces were integrated for 30.25 fs with a time step of 0.121 fs. This is a sufficiently long time, considering the 6.4-fs lifetime of the nitrogen core level.^52^ We assigned ammonia molecules in the dimer structures as donors or acceptors based on the starting geometries in the core-ionized MD. We first identified a hydrogen atom shared by both ammonia units by identifying the hydrogen with the minimal sum of distances to the nitrogen atoms. The ammonia molecule with its nitrogen atom covalently bound to this shared hydrogen is then designated as the hydrogen-bond donor.

Furthermore, we investigated the dynamics in the core-ionized state for larger ammonia clusters. The configurational space sampling was again performed with the PI+GLE approach. The simulation time step was again set to 20 a.u., and the overall simulation time was 15 ps. The temperature was set to 235 K as before. The forces for the ground-state MD simulations were obtained on-the-fly at the efficient BLYP/6–31G^**^ level with D3 correction^51^ by the TeraChem code. The simulated cluster contained 51 ammonia molecules. From the obtained geometries, 100 equally distanced frames were selected, and from each selected frame, 15 ammonia molecules were used for subsequent dynamics simulation in the core-ionized state. The dynamics in the core-ionized state was simulated with the wB97X/D functional and the cc-pVDZ basis set on the hydrogen atoms and the cc-pCVDZ basis set on the nitrogen atoms. The core-ionized ammonia molecule in the central position always acts as a proton donor.

The ground-state and core-ionized MD simulations were done with our in-house code ABIN,^53^ using the graphics processing unit (GPU)-based TeraChem program and the Q-Chem code version 5.4, respectively, for the calculations of energies and forces.^42,54–56^

## III. RESULTS AND DISCUSSION

### A. Experimental results

**FIG. 2.**
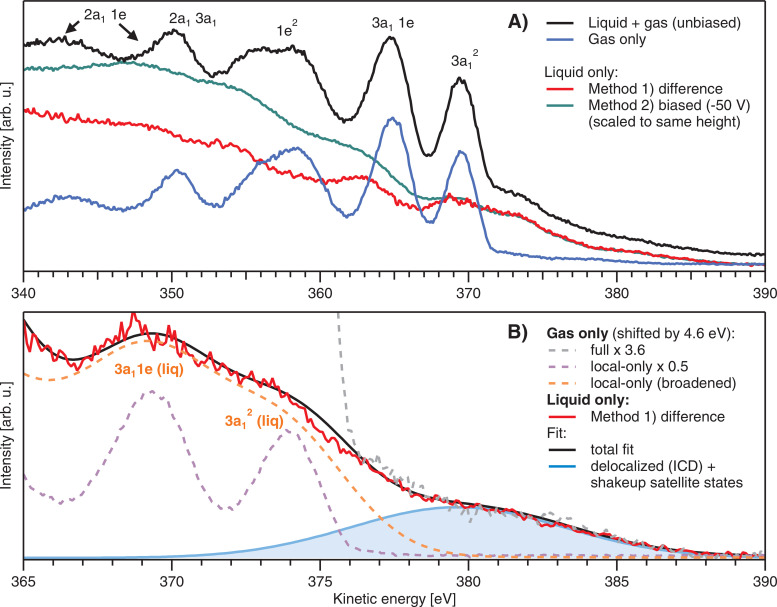
Auger–Meitner spectra from liquid- and gas-phase ammonia measured at a photon energy of 480 eV. (a) Overview over the full measured range. The spectrum measured from the grounded liquid jet is shown in black and contains signal contributions from both the liquid and gas phase, with the latter originating from the vapor layer around the LJ. Main Auger–Meitner gas-phase peaks are labeled according to Ref. 20. The blue line shows the pure gas-phase spectrum. Subtracting blue from black yields the red difference spectrum, which then contains only the liquid-phase signal. An alternative approach to remove the gas-phase contribution is to apply a large bias voltage to the LJ, which pushes the gaseous signal out of the energy region of interest (see the text for details). Such a biased spectrum is shown in green; here, the kinetic energy (KE) was corrected for the energy shift of 50 eV imposed by the bias voltage. We see an excellent agreement of spectral features from red and green, other than the difference in the broad background between 340 and 370 eV, which is due to residual, smeared-out gas-phase signal at lower KE. (b) Close-up of the high-KE region of (a). Here, we also present the gas-phase spectrum, multiplied by a factor of × 3.6 (grey) as compared to the case, where the main lines are matched to the liquid-phase spectrum to reveal the weak satellite structure at KEs between ∼378 and ∼387 eV. To model the liquid-only spectrum, we first prepared a gas-phase spectrum (purple), where the high-KE satellite structures have been subtracted (see Fig. S2 in the Supporting Information^81^). This gas-phase spectrum was shifted by 4.6 eV (= gas-liquid shift) and broadened with a Gaussian kernel of 2.65 eV width (unspecific intermolecular interaction), which yields the spectrum shown by the orange dashed line and mirrors the local Auger–Meitner peak structure well, i.e., features other than the additional contribution from high-KE states. Values for shifting and broadening were determined by fitting the red liquid spectrum (see Fig. S3 in the Supporting Information^81^), yielding a good overall fit (black). In the latter, a Gaussian (blue) was included to represent the associated signal from delocalized and satellite states near 380 eV.

Nitrogen 1s Auger–Meitner (autoionization) spectra from a liquid ammonia jet, measured at 480 eV photon energy from a grounded (black spectrum; method 1) and biased jet (green spectrum; method 2), respectively, are presented on an electron kinetic energy (KE) scale in Fig. 2(a). Spectra were obtained for the best overlap of x-ray focus and the LJ, which, in the case of method 1, maximizes the relative liquid-phase signal with respect to the gas-phase signal. The spectrum of method 2 (green) was measured for −50 V bias voltage, which energetically separates gas-phase and liquid-phase signal contributions, yielding a liquid-only spectrum essentially free of gas-phase signal in the high-KE region of interest (see Methods section). Here, the bias-induced shift in kinetic energy was subtracted, such that all spectra line up if measured at ground. We also present the gas-only spectrum (blue line) obtained when slightly moving the LJ out of the light focus, such that only the gaseous ammonia molecules surrounding the LJ were ionized; count rates were somewhat smaller due to the lower target density some distance away from the LJ and are scaled up to match the gas-phase signal in the black spectrum in the figure. Peak positions are in reasonably good agreement with reported gas-phase ammonia Auger–Meitner spectra.^20,57^ The peak assignments (labels in the figure) are taken from Ref. 20. The difference spectrum, black spectrum minus the scaled gas-phase spectrum (blue), also yielding a liquid-only spectrum, is shown by the red curve. The two liquid-phase spectra are scaled to match the intensity of the high-energy region, i.e., the part free of background-signal contributions. It is seen that even spectral structure details perfectly match, implying that both methods are equivalent; a detailed comparison is shown in Fig. S1 in the Supporting Information.3-5,^81^ This is an important result, which demonstrates that no spectral artifacts have been introduced into this region of ICD and satellite fingerprints. Larger background signal intensity at energies <370 eV for the biased jet is attributed to a residual broadened gas-phase contribution, and possibly to slightly different inelastic scattering contributions in the Auger–Meitner spectrum but neither effect matters for the present analysis, which focuses on the high-KE part of the spectra only. Note that the application of −50 V bias to a liquid water jet almost completely eliminates the gas-phase contributions from liquid-water spectra. This is due to the approximately 10-times lower vapor pressure/gas-density, surrounding the LJ in a typical liquid-water-jet photoemission experiment as compared to a liquid ammonia jet (considering vapor pressures of ammonia at –50 °C and water at room temperature, respectively).^37^ The higher ammonia vapor pressure also explains the very large gas-phase signal contribution in the Auger–Meitner spectrum from the grounded liquid jet (black line), amounting to approximately 65% of the total photoemission signal.

We next analyze the liquid-only N 1s Auger–Meitner/autoionization spectrum; see Fig. 2(b) for a close-up of the high-KE region. Our approach assumes that the main Auger–Meitner lines in the liquid-phase spectrum can be constructed from a shifted and Gaussian-broadened gas-phase spectrum, using the same orbital description. The validity of this approach — not accounting though for the non-local spectral features — has been recognized for liquid water,^58^ and more quantitatively demonstrated for ammonia clusters (mean size 1600 molecules/cluster).^23^ For this, we first construct a gas-phase spectrum with removed high-KE satellite features, which are separately accounted for in the following fit to the liquid-phase spectrum. The procedure for removing the satellite features is laid out in Fig. S2 in the Supporting Information;^81^ briefly, we fit a series of peaks to the as-measured gas-phase spectrum and subtract only the peaks associated with satellite features. The resulting satellite-free spectrum is shown as a purple dashed line in Fig. 2(b). A fitting algorithm is then employed to find the best overlap of this gas-phase spectrum with the liquid-only spectrum (red). The result (black curve) is shown in Fig. 2(b) (the full range of the fit is shown in Fig. S3 of the Supporting Information^81^). We find that the main (local) Auger–Meitner lines of the liquid-phase occur at approximately 4.6±0.3 eV larger KE than the respective gas-phase counterparts; the rather large error range is estimated as an upper limit to the influence of a possibly present residual streaming potential. This places the leading ammonia liquid-phase 3a_1_^2^ Auger–Meitner peak at 374 eV KE. The ∼4.6-eV energy shift can be attributed to screening by the polarizable environment. This is analogous to water, for which a similar KE shift of ∼4.5 eV was observed,^58^ despite the smaller electronic polarizability.^37^ A shift of 5.17 eV was observed theoretically (see Table S1 in the Supporting Information^81^) when going from the gas phase to the liquid phase modeled by a polarizable continuum. The aforementioned work on ammonia clusters estimated an approximately 6-eV energy shift in the Auger–Meitner spectra from observed gas–liquid shifts in the N 1s core level using Δ*E*_AM_ = 3Δ*E*_XPS_, which seems to be somewhat overestimated and may well be lower.^23^ The present value is directly inferred from both the experimental Auger–Meitner spectrum and theory and is, thus, arguably more reliable.

**FIG. 3.**
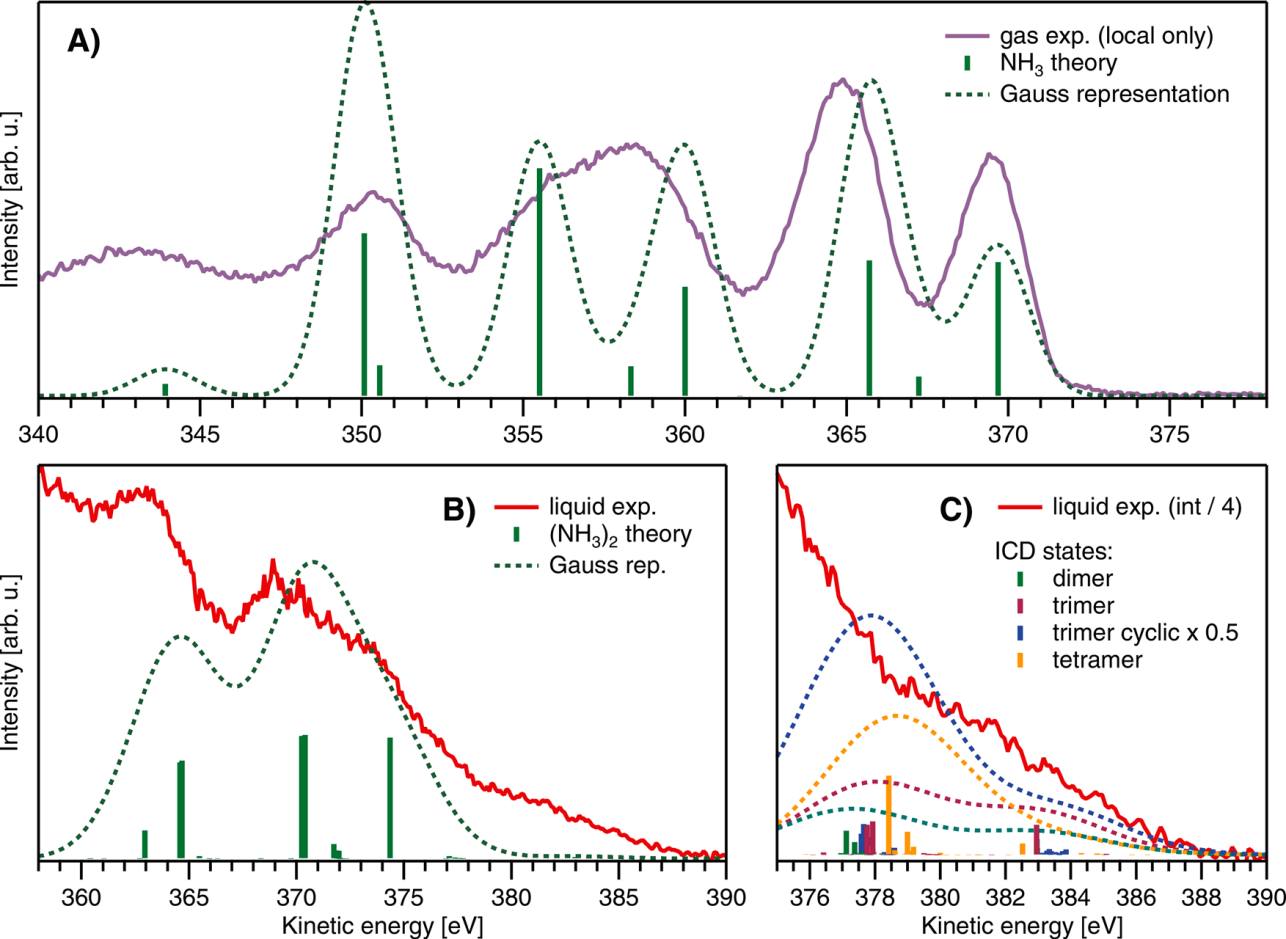
(a) Simulated N 1s Auger–Meitner lines for the gas-phase ammonia molecule and (b) for the optimized ammonia dimer at the EOM-CCSD level (green sticks), in comparison with the experiment (gas phase in purple and liquid in red). For the ammonia dimer, the N 1s ionization was localized on the proton donor. (c) Delocalized ICD states calculated at the EOM-CCSD level for the ammonia dimer, a random non-cyclic trimer and tetramer structure taken from the molecular dynamics simulation, and for an optimized global-minimum cyclic trimer structure. A Gaussian representation with a FWHM of 2.2 eV in (a) and 4.8 eV in (b) and (c) has been added as a visual aid. The scaling of the data vs ICD intensity is merely for a visual comparison and is not meant to imply the actual contribution of ICD to the overall high-KE signal. The results in B and C have been shifted by 3.7 eV toward higher KE to account for polarization screening. For the non-cyclic trimer and tetramer structures, the ICD intensity was calculated for the central ammonia molecule, and for the optimized cyclic ammonia trimer, all ICD states from all ammonia molecules are shown. The number of Auger–Meitner states for the ammonia dimer, trimer, and tetramer was limited due to convergence problems. Note that the shake-up states and shake-off states are not included in the present calculations.

More important, however, is the spectral region containing non-local signal contributions, occurring at KEs considerably above 374 eV (maximum of the leading Auger–Meitner peak), and extending to 388 eV. It is useful to first inspect the gas-phase spectrum in more detail because low-intensity gaseous spectral features do coincide with these high-KE liquid-phase features. This spectrum, reproduced in Fig. 2(b), has been shifted toward 4.6 eV higher KEs to align with the liquid-phase spectrum. We can observe that the gas-phase spectrum exhibits a signal contribution in the same 374–388 eV (after applying the shift) high-KE range of interest but has not been quantitatively addressed in any previous work. Yet, the occurrence of this structure has been mentioned before and only qualitatively attributed to satellites;^57^ it was not accounted for in the ammonia cluster study.^23^ Here, we provide an accurate interpretation, based on high-level electronic structure calculations, identifying these high-energy spectral contributions in the gas-phase spectrum as shake-up satellites. Another important observation from Fig. 2(b) is that this high-KE signal is larger by a factor of 3.6 in the liquid phase. We will argue in the following that satellite emission is not the main contribution to the liquid-phase spectrum in this high-KE region, and that a significant contribution also arises from non-local autoionization. Here, we have modeled the high-KE contribution, comprising both satellite and non-local contributions, with a single Gaussian (light-blue curve with the blue-shaded area) in our overall fit (black curve) in Fig. 2(b). Here, we include only the data from method 1 because the almost identical spectrum is obtained with method 2, as discussed above.

### B. Electronic decay processes in the gas phase: Theory

The theoretical gas-phase Auger–Meitner spectral lines modeled via the EOM-CCSD approach (green bars) are compared with the experimental gas-phase spectrum in Fig. 3(a); as a visual aid, the spectral lines are represented by a sum of Gaussians with a FWHM of 2.2 eV (green dashed lines). The N 1s core-level ionization energy at the CVS-EOM-CCSD/cc-pVTZ level is 405.96 eV (406.08 eV at CCSD(T)/cc-pVTZ and cc-pCVTZ level), which is in agreement with previous studies.^23^ The modeled spectra of the ammonia monomer capture some of the main spectral features; for instance, the peak position of the leading 3a_1_^2^ Auger–Meitner peak is very accurate. The energy of the 3a_1_ 1e peak is slightly overestimated by ∼1eV. Some of the channel rates (intensities) are overestimated, particularly high intensities are assigned to triplet channels at ∼350 and 355 eV, which is probably an artifact from using plane waves^41^ for the outgoing electron.

To provide further insight into the electronic decay processes leading to high-KE contribution in the gas-phase ammonia spectra, we estimated the energetics of all possible decay channels. We performed MOM-CCSD(T) and wB97X-D calculations for the leading Auger–Meitner peak as well as for the shake-up and shake-off processes. The investigated decay channels upon N 1s core ionization are schematically depicted in Fig. 1. The energies of the initial, intermediate, and final states are collected in Table S1 in the Supporting Information^81^ for the ammonia monomer. The KEs of the outgoing electrons were estimated as the energy difference between the intermediate and final states. For example, the kinetic energy of the electron arising from the shake-up intermediate state, decaying into a two-hole (2h) final state, was estimated as

$$KE_{\mathrm{shake}–\mathrm{up}}=E_{\mathrm{shake}–\mathrm{up}}-E_{2h}.$$

The respective KE values are provided in Table I. The resulting energies of the Auger–Meitner electrons for the gas phase agree well with the experiment, which shows that the high-KE tail in the gas-phase ammonia spectrum corresponds to decay from intermediate shake-up states. In contrast, the KEs corresponding to the intermediate shake-off states are about 20–25 eV smaller and are, thus, overlapping with the normal Auger–Meitner signal, which spans from 360 to 371 eV. Note that these channels will be diffuse due to the involvement of many different electronic states and possibly associated with the nuclear dynamics in the intermediate state.

**TABLE I.** Theoretically estimated Auger–Meitner-electron kinetic energies in eV for various decay channels of a core-level ionized ammonia molecule and for the ICD channel in the ammonia dimer and ammonia trimer (we assume that the proton donor is core ionized in the case of ammonia dimer and trimer taken from molecular dynamics, and in the case of optimized cyclic trimer, the core-ionized states are practically degenerate).

**NH_3_**				
Intermediate state	wB97X-D/pcm	CCSD(T)/pcm	wB97X-D/gas	CCSD(T)/gas
Core ionization (Auger–Meitner)	372.28	372.97	367.12	367.80
Shake-up	∼383	∼384	379.25	∼380
Shake-off	361.84	362.56	353.05	353.77
**NH_3_ ··· NH_3_**				
Intermediate state	wB97X-D/pcm	CCSD(T)/pcm	wB97X-D/gas	CCSD(T)/gas
Core ionization (ICD)	382.50	383.29	378.58	379.56
**(NH_3_)_3_ from dynamics**				
Intermediate state	wB97X-D/pcm	CCSD(T)/pcm	wB97X-D/gas	CCSD(T)/gas
Core ionization (ICD)	384.00	384.73	382.49	381.58
**(NH_3_)_3_ minimum**				
Intermediate state	wB97X-D/pcm	CCSD(T)/pcm	wB97X-D/gas	CCSD(T)/gas
Core ionization (ICD)	382.98	383.53	379.99	380.30

### C. Non-local electronic decay processes: Theory

Figure 3(b) shows the Auger–Meitner spectral lines calculated for the ammonia dimer (green bars); analogous to (a), a Gaussian representation with a FWHM of 4.8 eV (2.2 + 2.6 eV) is included as a visual aid (green dashed lines). All results for the comparison with the liquid phase [Figs. 3(b) and 3(c)] are additionally shifted by 3.7 eV to approximate the effect of polarization screening. When going from the monomer to the ammonia dimer, we observe a shift of the most intense Auger–Meitner peaks toward higher energies by ∼1 eV (i.e., 4.7 eV with the polarization shift) due to the solvation by the neighboring ammonia molecule. These Auger–Meitner peaks correspond to the situation when both the initial core ionization and final 2h states (doubly ionized states) are localized on the same subunit (in our case on the proton donor unit of the dimer). The N 1s core-ionization energy at the EOM-CCSD/cc-pVTZ level is 405.20 eV (the same value is obtained at the CCSD(T)/cc-pVTZ and cc-pCVTZ level), which corresponds to a chemical shift of ∼0.7 eV. The number of Auger–Meitner lines is higher for the ammonia dimer, and most importantly, there appear spectral features at energies >376 eV, which correspond to delocalized final states, i.e., the ICD states. Figure 3(c) shows a close-up on these ICD states, with additional results for the ammonia trimer and the tetramer. The appearance of these delocalized states is in agreement with previous algebraic diagrammatic construction (ADC) calculations by Kryzhevoi and Cederbaum.^25^ The modeled intensity of the ICD states is low, an order of magnitude lower than the intensity of the leading 3a_1_^2^ Auger–Meitner emission. However, the intensity of these states will be higher when taking into account that the number of neighboring molecules is higher in liquid ammonia. As is apparent in Fig. 3(c), the intensity of the ICD signal increases for the ammonia trimer and the tetramer. According to the structural properties studied by neutron diffraction,^59–62^ elastic^63,64^ and inelastic x-ray scattering^65^ experiments, and theoretical simulations,^66–70^ the coordination number in liquid ammonia is ∼12–14, and the first solvation shell is much less structured compared to the solid phase.^71–73^ We can, thus, expect a further increase in the ICD signal when extrapolating our observations to the liquid phase.

The KE of the ICD electron was estimated for the ammonia dimer and trimer both in the gas and liquid phases; resulting values are provided in Table I (a more detailed list can be found in Tables S2 and S3 in the Supporting Information^81^). According to the calculations, the ICD electrons are predicted to appear at around 380 eV for the gas-phase dimer and around 383 eV for the liquid-phase dimer and trimer (at both wB97X-D and CCSD(T) levels). These energies coincide with the high-kinetic energies in the experimental spectrum (Fig. 2).

### D. Does proton transfer contribute to the high-energy peak?

**FIG. 4.**
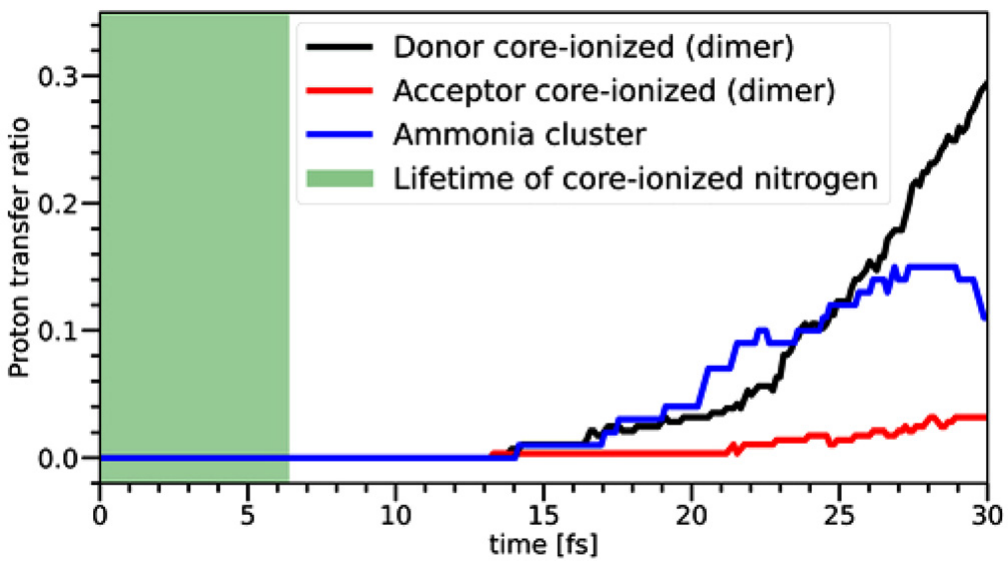
Time evolution of structures with a proton transfer following the core ionization of ammonia. Results for a gas-phase dimer (black and red) and ammonia cluster (blue) are shown. No proton transfer is observed within the nitrogen core-hole lifetime (shown by the green shading). We evaluated the proton transfer based on the distances between all hydrogen atoms (nuclei) surrounding the core-ionized ammonia and all nitrogen atoms. The proton transfer was considered to take place when the number of hydrogen atoms closest to the core-ionized ammonia was different than three. MD simulations in the core-ionized states of the dimer were carried out with the wB97D functional, using the cc-pCVTZ basis set on the nitrogen atoms and the cc-pVTZ basis set on the hydrogen atoms. For larger clusters, the cc-pCVDZ basis set was used for the nitrogen atoms and the cc-pVDZ basis set for the hydrogen atoms.

We explored other possible origins of the high-KE peaks in the Auger–Meitner spectrum using *ab initio* simulations. First, the role of proton transfer (PMT-CS) upon the core-level ionization of an ammonia dimer in the gas phase was investigated. We performed semiclassical adiabatic simulations of the first 30.25 fs following N 1s ionization. The initial structures were taken from PI+GLE simulations; here, we considered the ionization of either proton donor or proton acceptor (see Methods section for details). The simulations indicate that no proton transfer takes place within the first 10 fs in both cases. After 20 fs, the probability of proton transfer remains negligible when the proton acceptor is ionized, and it amounts to approximately 4% when the proton donor is ionized (see Fig. 4). The short-time dynamics is dominated by the intramolecular umbrella-type motion of one of the ammonia units. In addition to the ammonia dimer, the dynamics on the core-ionized state for clusters containing 15 ammonia molecules was also simulated for the first 30 fs following the N 1s core ionization of the central ammonia molecule. The initial structures were taken from the PI+GLE simulations for a cluster of 51 ammonia molecules; the central molecule always assumes the position of the proton donor. The general trend is very similar to the observations for the water dimer. Based on this result, we conclude that the proton-transfer reaction is quite limited within the N 1s core-hole lifetime of 6.4 fs.^52^ In the simulations, we also observe a substantial planarization of the ammonia molecule, which is consistent with previous findings for the isolated ammonia molecule and ammonia solvated with water.^9^

**FIG. 5.**
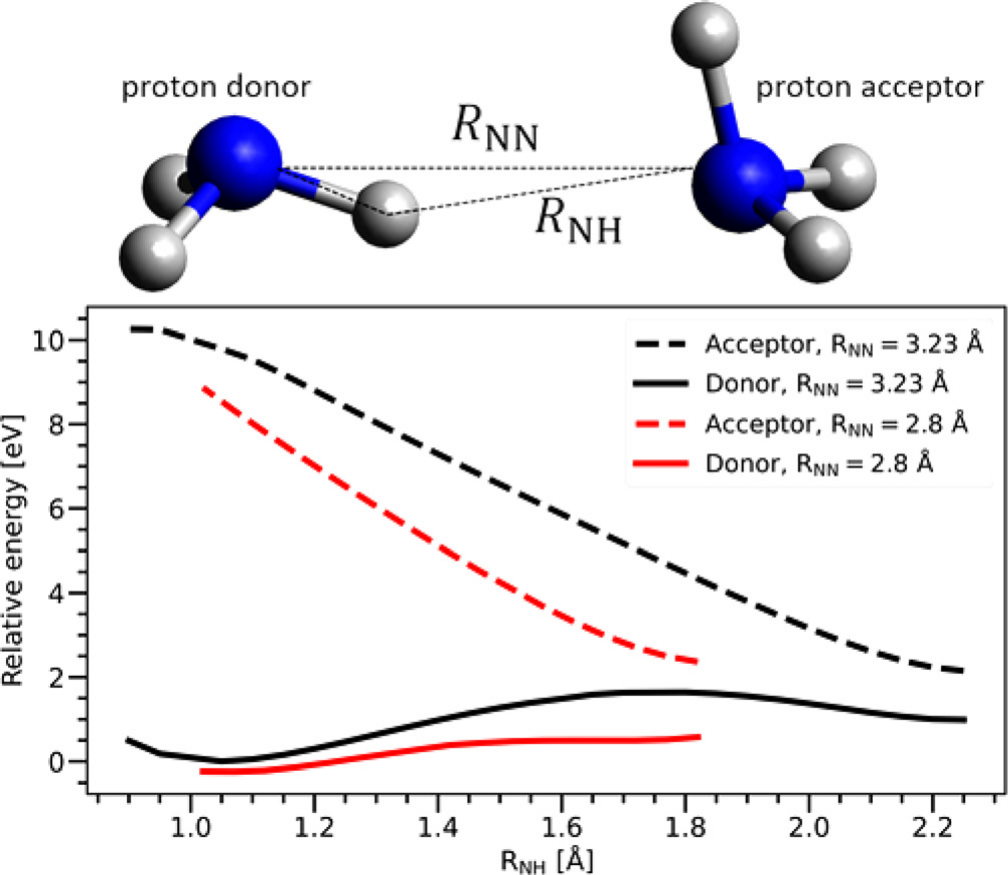
Calculated energy curves for a core-ionized ammonia dimer along the proton transfer coordinate. We assumed two geometries: equilibrium geometry of the ammonia dimer (black) and a geometry with an artificially shortened distance between the two nitrogen atoms (red). The latter distance was set to 2.8 Å, representing the onset of the radial distribution function. Energies are shown as a function of the distance between the acceptor nitrogen atom and the proton. The energies were calculated with the MOM method at the CCSD(T) level and with the cc-pCVTZ basis set on the nitrogen atoms and the cc-pVTZ basis set on the hydrogen atoms. The optimal structure for the ammonia dimer was taken from Ref. 79.

The results of the dynamical calculations (Fig. 4) are consistent with the static *ab initio* calculations shown in Fig. 5, indicating that there is a relatively high energy barrier (on the order of tenths of eV) for the proton transfer for the equilibrium geometry of the ammonia dimer. Figure 5 also shows that the barrier decreases with the distance between nitrogen atoms similar to previously reported ammonia dissolved in water.^9,74^ In contrast, for water, the O–O distances are shorter in the liquid phase than in the gas-phase dimer. However, both experiment and previous simulations as well as our new MD simulations show that the N–N distance in liquid ammonia is larger than in the gas-phase dimer, which hampers the proton transfer. Specifically, the maximum of the first peak in the N–N radial distribution function (RDF) is found at 3.16 Å in our MD simulation of the ammonia dimer, and at 3.28 Å in the MD simulation of the ammonia cluster containing 51 ammonia molecules. The N–N distance in the gas-phase dimer is 3.23 Å.^74^ For bulk systems, the first maximum of the RDF by x-ray diffraction experiment is 3.37 Å (see Ref. 75 and references therein), while polarizable MD simulations and *ab initio* MD provide 3.50 Å^75^ and 3.45 Å,^29^ respectively. Furthermore, it was previously reported that the proton transfer depends on the orientation of the hydrogen bond,^9^ which, in the case of ammonia, deviates significantly from collinearity. Overall, the only factor that could, in principle, favor proton transfer in ammonia is the increased N 1s core-hole lifetime of ∼6.4 fs compared to the O 1s lifetime of ∼4 fs (Ref. 76) in water. This, however, is not sufficient for a substantial proton transfer, as explained above. These findings confirm the importance of hydrogen-bond strength for PTM-CS to occur, and our studies suggest similar (small) strengths in pure liquid ammonia (NH_3_ – NH_3_) and the aqueous environment (NH_3_ – OH_2_), resulting in similarly low probabilities for PTM-CS. In other words, PTM-CS can serve as a measure of the hydrogen-bond strength.

### E. Interpretation of high-KE region of the Auger–Meitner spectrum

In the following, we summarize our experimental and computational findings and discuss their implications for the high-KE signal of the liquid-phase Auger–Meitner electron spectrum in the 375–385 eV kinetic-energy range. Note that the fast planarization of the ammonia molecule upon core-level ionization affects the gas-phase and the liquid-phase Auger–Meitner spectra similarly and is not further discussed here. We then consider three possible sources of fast electrons.

#### 1. Electronic decay assisted by nuclear dynamics

**FIG. 6.**
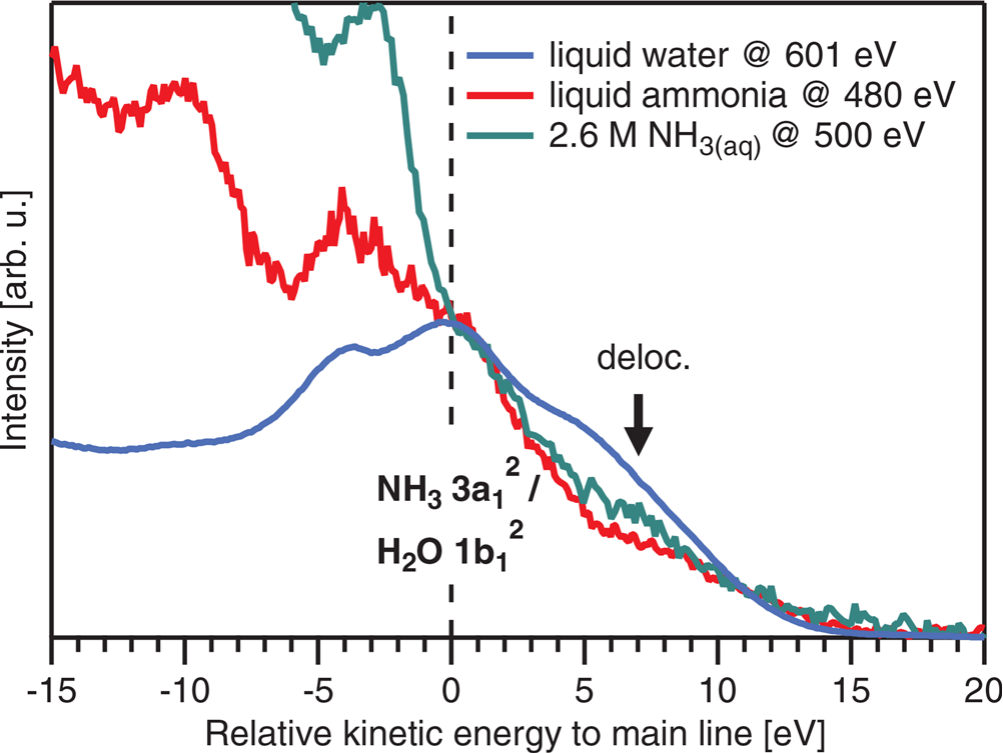
Auger–Meitner spectra with ICD contributions from liquid ammonia (red), liquid water (blue), and ammonia in water (green; from Ref. 9). The KE energy scale is presented as relative KE with respect to the position of the leading local Auger–Meitner peak, 3a_1_^2^ of ammonia and 1 b_1_^2^ for water. Intensities are scaled such that these leading peaks (at relative KE = 0 eV) of the three solutions have the same peak height.

The *ab initio* simulations indicate that the PTM-CS process is not relevant for liquid ammonia where hydrogen bonding is weaker than in liquid water. This implies that the electronic decay occurs from the structure close to the ground-state geometry. This conclusion is further confirmed by the comparison of the present Auger–Meitner spectrum with the spectrum of the liquid water, for which the PTM-CS process is well established.^1^ In water, the PTM-CS component appears in the Auger–Meitner spectrum at energies about 5 eV above the leading regular Auger–Meitner peak. Also, the exact energy of the final states of PTM-CS-induced transient structures depends on proton separation distance, and the PTM-CS peak is manifested by a high-energy shoulder of the leading peak of (local) Auger–Meitner decay. If the proton transfer does not take place, the energy shoulder should be weak, and rather, an isolated ICD peak appears. Experimentally, we observe a considerably smaller shoulder as compared to liquid water, accompanied by a small feature at approximately 7 eV higher KE than the leading Auger–Meitner peak (Fig. 6), which, indeed, seems to indicate the presence of an isolated ICD feature.

#### 2. Purely electronic intermolecular Coulombic decay

This non-local process is likely significantly contributing to the signal in the high-KE region. The high-kinetic energy component of the spectrum has, however, a surprisingly large intensity, at least 6.6% (compare Fig. S3), well exceeding a 1% effect observed in a recent core-level ICD study from argon clusters;^11^ we are not aware of any other related rare-gas core-level study. The theoretical calculations for the ammonia dimer predict a much smaller contribution from the ICD channel, but calculations for larger clusters support the expected growth of this channel extrapolated to the condensed phase. Potentially, an experimental identification of ICD could be obtained by ionizing ammonia closer to the N 1s edge, i.e., at energies where the shake-up channels are still closed. Another, much more challenging approach may be electron–electron-coincidence measurements. Both experimental routes are not feasible for the foreseeable future.

#### 3. Shake-up processes

We have shown that the satellite features associated with valence-excitation processes energetically overlap with the ICD signal, and that they are the sole origin of the high-KE signal in gas-phase ammonia Auger–Meitner spectra. It is conceivable that the intensity of shake-up channels can be enhanced in the condensed phase due to an increased number of available final states, e.g., via non-local processes. At this moment, we cannot separate the contributions from the ICD and non-local shake-up processes. Such calculations are beyond state-of-the-art capabilities. However, the nearly fourfold increase in signal strongly suggests that shake-up processes alone cannot be the sole origin of this signal, and compared to our calculations and the model case of liquid water, we argue that a significant contribution comes from purely electronic ICD.

We note that the ammonia-cluster study of Ref. 23 reports a spectrum quite similar to the liquid-ammonia spectrum (red), albeit lacking the pronounced inelastic scattering background characteristic for a bulk liquid. A similar observation was made for liquid water, where, in contrast to water clusters, a strong inelastic background signal is present.^77^ The similar shape of the cluster and liquid ammonia spectra, and even of the amorphous solid state of ammonia, may suggest that pure electronic decays, not accompanied by proton dynamics, occur in all these systems.

## IV. CONCLUSIONS

This work reports on the first Auger–Meitner electron spectrum of liquid ammonia upon N 1s core-level ionization. Because of ammonia's high vapor pressure, the acquisition of Auger–Meitner spectra was complicated by a high density of gas-phase ammonia, much higher than typically encountered in a liquid-jet photoemission experiment from aqueous solutions. Nevertheless, our measurements allowed for an unambiguous extraction of the pure liquid-phase Auger–Meitner spectrum, and spectral assignment with a particular emphasis on associated non-local autoionization processes.

The leading Auger–Meitner electron peaks in liquid ammonia are shifted by ∼4.6 eV toward higher kinetic energy relative to the peaks of gas-phase ammonia. A shift of ∼4.5 eV has also been observed for liquid water,^58^ in agreement with the similar size of the molecules and the similar electronic polarizability of both liquids. The shift is also similar to the value observed in solid-state ammonia.^24^ Note that in the solid state, the absolute value of the shift cannot be inferred since the work function of the solid is not known, and, thus, a direct comparison with the gas phase is impossible. Analysis of cluster spectra is complicated by the presence of monomers in the supersonic beam. The energy shift measured in the present work provides a first direct estimate of the polarization effects on the Auger–Meitner spectrum in liquid-phase ammonia.

A pronounced signal at higher kinetic energies, separated by ∼7 eV from the leading Auger–Meitner peak, in the case of liquid ammonia is partially attributed to shake-up processes since a similar, but weaker, signal is observed even for the isolated ammonia molecule in the gas phase. In the liquid phase, the signal in that region is larger by a factor of about 3.6. We attribute this additional signal to the pure electronic decays, not accompanied by intermolecular nuclear dynamics. Contribution from ICD is supported by calculations with the Fano–Feshbach theory using the EOM-CC framework, with increased ICD intensity in the tetramer relative to the trimer, and the trimer relative to the dimer, respectively. Simulations for larger clusters remain out of reach at the moment. We expect further increase in the liquid phase. Yet, we cannot rule out the possibility that the shake-up process might be somewhat enhanced as well. Despite the relatively long N 1s core-hole lifetime (6.4 fs), the probability of PTM-CS processes is very small.

Liquid ammonia is a hydrogen-bonded system, and the electronic processes can couple to nuclear dynamics following core-level ionization. In agreement with previous works, we observed in our MD simulations a fast umbrella-type motion. Unlike in liquid water, we find no significant proton-transfer dynamics occurring between the hydrogen-bonded units within the respective N 1s core-hole lifetime. This shows that spectroscopic identification of the relative strengths of non-local electronic processes vs PTM-CS assists in characterizing the hydrogen-bond strengths of solutions in general.

## Data Availability

The data that support the findings of this study are openly available in Zenodo at http://doi.org/10.5281/zenodo.6509532, Ref. 78.
